# An evaluation of the Kodak glucose/ BUN analyser including experience with proposed testing protocols

**DOI:** 10.1155/S1463924681000497

**Published:** 1981

**Authors:** H. M. Barbour, K. Virapen, T. F. Woods, D. Burnett

**Affiliations:** Departments of Clinical Biochemistry Queen Elizabeth II Hospital Welwyn Garden City and St. Albans City Hospital St. Albans, Herts UK


### An evaluation of the Kodak glucose/ BUN analyser including experience with proposed testing protocols

**H.M.Barbour, K. Virapen, T.F. Woods and D. Burnett**

*Departments of Clinical Biochemistry, Queen Elizabeth II Hospital, Welwyn Garden City and St. Albans City Hospital, St. Albans, Herts, UK.*

### Introduction

The Eastman Kodak Company has recently described a new concept in clinical chemistry, namely the use of dry chemistry films to be used initially for colorimetric analysis [2,3]. The glucose and urea methods were evaluated in this study following the guidelines by the National Committee for Clinical Laboratory Standards (NCCLS) [1] which are recommendations for goals to be sought by manufacturers. This evaluation protocol is designed to provide a multi-purpose evaluation framework for a wide range of methods and instruments and is in three sections. PSEP-2 (proposed standard for establishing performance claims) describes the four-week baseline period which is used to establish confidence limits for the controls used throughout the study. PSEP-3 details the precision study and PSEP-4 describes the comparison of methods experiment.

The Kodak Ektachem GLU/BUN analyser is a micro-processor controlled discrete analyser which operates in single or dual test mode. The instrument used in the present study was an engineering model used in the USA and Europe and was designed to evaluate the concept of quantitative chemical analysis using multilayered reagents. It was used according to the operators manual. The instrument was modified to give urea values, as opposed to blood urea nitrogen values, and was calibrated in mmol/L.

### Methods and materials

**NCCLS protocol for establishing performance claims for clinical chemistry methods**

The protocol is in three sections [1]. The sections and the material used are described below.

*Performance check experiment PSEP-2*

The protocol describes control criteria which were established for the test method. These criteria were used to control performance of the Kodak test methods during the performance of subsequent sections of the protocol. The control sera used were lyophilised human material provided by Kodak for the LOW and HIGH levels and Wellcomtrol II (Wellcome Reagents Limited, Kent, UK) for the MID-level.

*Replication experiment PSEP-3*

The replication (imprecision) protocol specifies a period of twenty days and a total of forty analytical runs. The 'midi' version was chosen as it is designed for medium rate automated methods. The midi experiment involves the analysis of half the number of samples compared with the maxi version, and unlike the mini experiment still enables the effects of carryover to be investigated. For each concentration level studied two different estimates of within run and total imprecision are required for presentation of performance claims. The first, designated 'point estimate', is the actual standard deviation observed in the experiment performed and the second, designated 'tolerance limit', represents the upper limit that with 95% confidence will contain the estimate of standard deviation from 99% of all similar experiments.

The replication experiment used the three controls from the performance check period together with Pathonorm L (PATH-L) from Nygaard, BDH, Poole, England; Wellcomtrol I (WELL-I) from Wellcome Reagents Limited, Beckenham, England and GEO A632 from General Diagnostics, New Jersey, USA. Lyophilised material was stored at 4°C prior to reconstitution on the morning of each experimental run.

*Comparison of methods experiment PSEP-4*

In this experiment a series of patient samples are analysed by both the test method and a comparative analytical method. A suggested concentration distribution of samples for a number of analytes is given in the protocol. Table 1 gives the distribution suggested by the NCCLS in PSEP-4 and the actual distribution used for urea and glucose samples.

Patient samples were collected over four weeks prior to the comparison of methods experiment. Serum samples for urea and plasma samples (fluoride-oxalate anticoagulant) for glucose were collected and stored at -20°C. Each day twenty-five samples were thawed and analysed as random duplicates by the test and comparative methods. Analyses were performed within two hours by both techniques, and each set contained samples in all category levels.

Regression analysis on the data for each analyte used the mean of paired duplicates. The comparative method was the independent variable (X) and the Kodak Ektachem system the dependent variable (Y). In order to illustrate the effect of the advice given in the NCCLS protocol on preparation of manufacturers claims three sets of data have been used for regression analysis. Set $e_1$ included results from all samples. Set $e_2$ excluded samples if the mean of any duplicate by the Kodak Ektachem system was outside the dynamic range specified in the operators manual [4], that is for glucose 1.11-33.3 mmol/l and for urea 0.71-42.8 mmol/l, or if the mean of any duplicate by the comparative method was outside the manufacturers quoted dynamic range.

Set $e_3$ was prepared by using the standard error about the regression line (Syx) calculated from data set $e_2$ to apply the test for outliers whereby up to three pairs showing a difference of greater than 3.5 times Syx can be excluded before the final regression analysis. The number of pairs with a difference in excess of 3.5 times Syx is given with all regression statistics. The regression statistics obtained on data set $e_3$ were used to calculate average bias (Yc-Xc, where Yc is the test method value at medical decision concentration Xc) at different medical decision concentrations. Tolerance limits were calculated for Yc so that there is a 99% probability that 95% of the sample results are included within the upper and lower limits and total error was established as the absolute value of the largest difference between the tolerance limits and Xc. It is recommended that the tolerance limits and total error be calculated only for the medical decision concentration closest to the mean of the comparative method results. Evident non-linearity of the data must be assessed visually in a scatter plot. It has been recommended [5] that the linear regression procedures used in this paper should be restricted to those cases where the correlation coefficient (r) exceeds 0.99. Similarly it has been suggested [6] that the range of values used is adequate when the standard deviation of the comparative method values (SDx) is greater than seven times Syx. The correlation coefficient and the ratio SDx/Syx are related [7]. With a slope of 1.00 and when the number of pairs is 200 and SDx/Syx = 7.0 then r = 0.9900. Values for both r and SDx/Syx are given with regression statistics. The relationship between the two values is such that changes in SDx/Syx are easier to observe.

**Wellcome Group quality control programme**

Samples previously sent out by the scheme from 16th October 1978 to 26th March 1979 were analysed by the comparative and Kodak methods. Two duplicate sets of twelve lyophilised bovine sera were provided and a sample for analysis taken from each of the twenty-four bottles after reconstitution, giving twelve duplicate analyses for each analyte.

Analysis of the twelve samples by participating laboratories is normally spread over a six month period and the analysis of results returned includes the overall mean for each analyte, that is the mean of all results returned, with results greater than three standard deviations from the mean excluded, and method means which represent the mean of all results from laboratories with a particular method classification. Appropriate standard deviations are also provided. The overall mean values in the samples used ranged from 3.40 to 13.77 mmol/L for glucose and from 5.21 to 23.61 mmol/l for urea.

**Comparative analytical methods**

Standard AutoAnalyzer I (Technicon) methodologies were used as the comparative methods. The diacetyl monoxime reaction for urea employed aqueous urea standards, the sample rate was 60/hour and samples with values above 20 mmol/l were diluted one in five in deionised water. For glucose the method was a glucose oxidase/peroxidase reaction with phenol and aminoantipyrine. Standards were prepared in saturated aqueous benzoic acid solution, the sample rate was 60/hour and sera with values above 20 mmol/l were diluted one in five in deionised water.

**Kodak methods**

Details of the glucose and urea slide chemistry are described by Curme *et al* [2] and Spayd *et al* [3]. Cartridges containing fifty slides were stored at 4°C and allowed to warm to room temperature for half an hour before the foil pack was opened. All experimental work reported here employed one coating batch, with daily calibration using three serum calibrators. Two hundred microliter samples were used for all studies. Samples with urea values above 40 mmol/l were diluted one in two with water.

### Results

**Performance check experiment (PSEP-2)**

Baseline performance data for forty sets of triplicate determinations over a twenty day period appear in Table 2, which provided performance check parameters for the replication and method comparison studies. Forty consecutive sets of readings were found using the criteria in the protocol.

Performance during the rest of the study was assessed by the mid control charts as described in the NCCLS protocol. Charts were constructed for the high, mid and low controls. The high and low control charts were only used as corroborative evidence if an outlier occurred in the mid level charts. Throughout the study there was only one mid control outlier. One reading of a triplicate set produced a mean and range error. However, the high and low control mean and range charts were well within limits and so this run was not rejected.

**Replication experiment (PSEP-3)**

Table 3 gives the analysis of variance (ANOVA) results for the replication experiment. It should be noted that the carryover effect on WELL-I for glucose and urea is based on testing significant differences in variance with and without

**Table 1. Distribution of samples for comparison of methods experiment**

**Glucose**

| Categories | A | B | C | D | E |
|---|---|---|---|---|---|
| Range (mmol/l) | <2.8 | 2.9-6.1 | 6.2-8.3 | 8.4-13.8 | >13.8 |
| Suggested sample distribution (%)* | 10 | 40 | 30 | 10 | 10 |
| Number of samples | 20 | 80 | 40 | 20 | 40 |
| % distribution achieved | 10 | 40 | 20 | 10 | 20 |

**Urea**

| Categories | A | B | C | D | E |
|---|---|---|---|---|---|
| Range (mmol/l) | <5.3 | 5.4-8.9 | 9.0-17.9 | 18.0-35.6 | >35.6 |
| Suggested sample distribution (%)* | 20 | 40 | 20 | 10 | 10 |
| Number of samples | 40 | 80 | 40 | 20 | 20 |
| % distribution achieved | 20 | 40 | 20 | 10 | 10 |

**NCCLS PSEP-4. See text for discussion of glucose distribution*

carryover. Table 4 gives the claims for imprecision with and without carryover following the NCCLS format.

**Comparison of methods experiment (PSEP-4)**

Figures 1 and 2 give the regression statistics together with correlation coefficient and the ratio SDx/Syx for patient samples used in the comparison of methods experiment. The preparation of data sets $e_1$, $e_2$ and $e_3$ is described in the Methods and Materials section. Regression statistics from data set $e_3$ were used to calculate the accuracy performance claims given in Table 5.

**Wellcome Group quality control programme**

There were two main objectives in using the multi-level lyophilised material available from the programme:

1. To provide additional information to that obtained in the comparison of methods experiment and compare the estimates from regression analysis with those calculated from patient samples. No exclusion criteria were applied to these results as the number of samples was very small. The ratio SDx/Syx in every case was well in excess of 7.0 and no pairs showed a difference in excess of 3.5 times Syx (Table 6 lines (a) and (e)).
2. In studies involving comparison of methods, conclusions concerning the performance of the test method are very dependent on the performance of the comparative or reference methods. Each comparative method can be classified in the Wellcome Scheme and the results obtained in each laboratory evaluated against the method mean (Table 6 lines (c) and (g)). Additionally they can be evaluated against the overall mean (Table 6 lines (d) and (h)).

The Kodak results are evaluated against the overall mean as it is difficult to classify this methodology in the Wellcome Scheme method classification (Table 6 lines (b) and (f)).

### Discussion

The establishment of performance and claims for clinical chemical methods has now become a major problem for manufacturers of clinical chemistry systems and is in danger of consuming a major part of a limited resource, namely that of skilled laboratory workers.

In these activities however there are complex problems for manufacturers and clinical chemistry laboratories alike and the publication of proposed standards PSEP-2, 3 and 4 by the NCCLS represents an important contribution to progress in this field.

This paper and the subsequent one [8] reports some of the authors' experience with these standards and the data derived from their work.The PSEP-2 and 3 standards although time consuming present few difficulties in execution based as they are on freeze dried material. However there are difficulties in carrying out the proposed standard for the Comparision of Methods Experiment (PSEP-4). The difficulties relate on the one hand to the selection of patient samples and their analysis according to the protocol and on the other hand to the performance of the comparative methods during the period of study.

The overview of the Comparison of Methods Experiment suggests that "at least 100 fresh patients' samples should be analysed in duplicate by both the test method and the comparative analytical method. The experiment must cover a period of at least four days which permits a maximum of twenty-five samples to be analysed in one day, or it can

**Table 2. Performance check period (PSEP-4) statistics**

| | Controls | | |
|---|---|---|---|
| | Low | Medium | High |
| **Glucose** | | | |
| $\bar{\bar{x}}$ Grand mean | 3.390 | 11.590 | 28.530 |
| Standard deviation (SD) | 0.062 | 0.198 | 0.304 |
| Control limits ± 3 x SD | 0.186 | 0.594 | 0.912 |
| Mean range $\bar{R}$ | 0.084 | 0.193 | 0.321 |
| Control limits 2.57 $\bar{R}$ | 0.216 | 0.496 | 0.824 |
| **Urea** | | | |
| $\bar{x}$ Grand mean | 3.170 | 29.130 | 36.550 |
| Standard deviation (SD) | 0.145 | 0.512 | 0.877 |
| Control limits ± 3 x SD | 0.435 | 1.540 | 2.63 |
| Mean range $\bar{R}$ | 0.080 | 0.596 | 0.828 |
| Control limits 2.57 $\bar{R}$ | 0.206 | 1.530 | 2.130 |

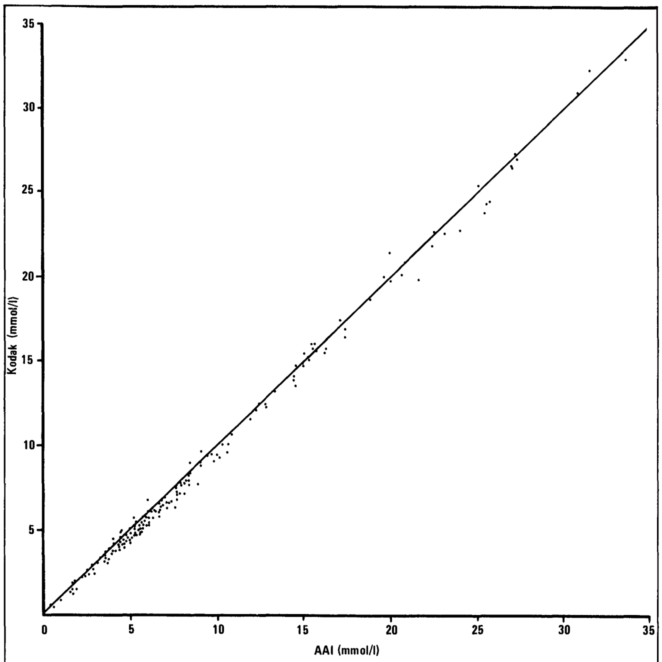

*Figure 1. Glucose Comparative Method (X) and Kodak Ektachem (Y) for patient samples.*

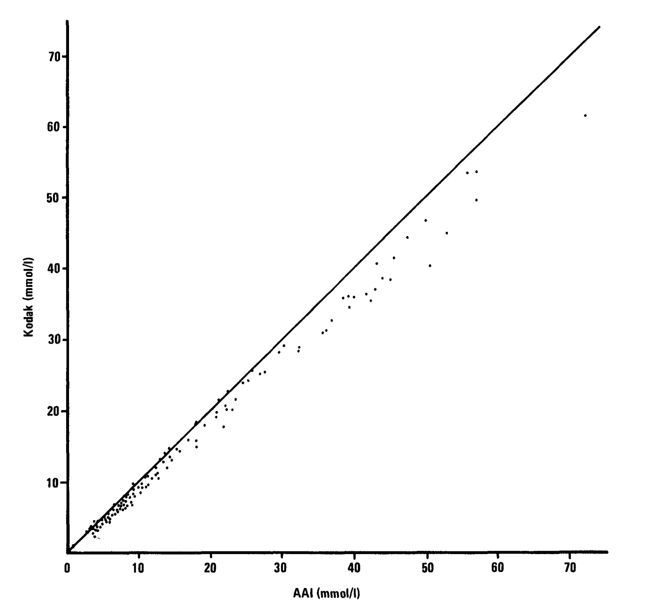

*Figure 2. Urea Comparative Method (X) and Kodak Ektachem (Y) for patient samples.*

extend over a longer period of time if that is convenient for the evaluation study". Recommendations for the selection of patients samples are given and one suggested distribution is shown in Table 1.

The authors were only able to comply with the suggested distribution by preselecting samples and freezing them prior to subsequent duplicate analysis. The amount of sample required to perform duplicate analysis by the test and comparative method represents a major problem if the test and/or comparative methods require substantial amounts of serum or plasma. The use of the NCCLS protocol for evaluation of multichannel systems may present special difficulties although one such evaluation has recently been published [9].

A penalty of not running duplicates is the failure to produce within run estimates of imprecision for human sera for the test and comparative methods. The information is important for evaluation of the comparative method and for its comparison with the test method. Additionally these estimates of within run imprecision can be usefully compared with those obtained in the replication experiment using the lyophilised material.

It was found that there was a tendency when selecting samples for analysis to encounter difficulties at the ends of the range. This could lead to the multiple selection of samples from one patient so that although the number of samples required is fulfilled the variability represented by those samples is reduced. If this were to become a major feature of selection then it might result in falsely low estimates of Syx which would markedly improve the accuracy performance claims. It is interesting in this connection to compare the regression statistics for comparison of methods for the bovine material from the Wellcome Scheme (Table 6 lines (a) and (e)) with those obtained on patient samples (Figures 1 and 2). Bearing in mind the recognised problems associated with commutability of samples and the small number of Wellcome samples used the estimates of slope and intercept were in agreement with those obtained with patient samples. However the value for Syx for urea and glucose using Wellcome material is markedly lower than the value for patient samples. This reflects the fact the Wellcome material is taken from only four homogenous pools and covers a smaller range of analyte concentrations.

If in excess of fifty quality control samples were used in a comparison of methods experiment and commutability were satisfactory the standard deviation of the estimates of slope and intercept would be markedly improved but analysis of lyophilised material from different sources can never replace patient samples in estimation of the standard error of the regression line.

**Table 4. Performance claims for imprecision with and without carryover**

| Serum | Concn mmol/l | Within run imprecision SD | Within run imprecision Tolerance limit | Total imprecision SD | Total imprecision Tolerance limit |
|---|---|---|---|---|---|
| **Glucose** | | | | | |
| Without carryover | | | | | |
| Path-L | 1.465 | 0.0226 | 0.0307 | 0.0735 | – |
| Well-I | 6.257 | 0.0868 | 0.1180 | 0.1204 | – |
| Geo A632 | 26.068 | 0.1834 | 0.2494 | 0.4637 | – |
| With carryover | | | | | |
| Path-L | 1.470 | 0.0253 | 0.0344 | 0.0747 | 0.0964 |
| Well-I | 6.293 | 0.1296 | 0.1762 | 0.1712 | 0.2208 |
| Geo A632 | 26.063 | 0.1579 | 0.2147 | 0.4547 | 0.5866 |
| **Urea** | | | | | |
| Without carryover | | | | | |
| Path-L | 2.961 | 0.0378 | 0.0514 | 0.1210 | – |
| Well-I | 9.095 | 0.1215 | 0.1652 | 0.1920 | – |
| Geo A632 | 31.761 | 0.4851 | 0.6597 | 0.5561 | – |
| With carryover | | | | | |
| Path-L | 2.956 | 0.0327 | 0.0445 | 0.1214 | 0.1566 |
| Well-I | 9.069 | 0.1842 | 0.2505 | 0.2469 | 0.3185 |
| Geo A632 | 31.777 | 0.4856 | 0.6604 | 0.5439 | 0.7016 |

**Table 3. Analysis of variance (ANOVA) of data from four week precision study with and without effects of carryover**

| | Conc mmol/l | Within run SD | Within run CV (%) | Within run (df) | Run-run SD | Run-run CV (%) | Run-run (df) | Day-day SD | Day-day CV (%) | Day-day (df) | Total SD | Total CV (%) | Total (df) |
|---|---|---|---|---|---|---|---|---|---|---|---|---|---|
| **Glucose** | | | | | | | | | | | | | |
| Without carryover | | | | | | | | | | | | | |
| Path-L | 1.465 | 0.0226 | 1.54 | (80) | 0.0116 | 0.79 | (20) | 0.0690 | 4.71 | (19) | 0.0735 | 5.02 | (119) |
| Well-I | 6.257 | 0.0868 | 1.39 | (80) | 0.0233 | 0.37 | (20) | 0.0800 | 1.28 | (19) | 0.1204 | 1.92 | (119) |
| Geo A 632 | 26.068 | 0.1834 | 0.70 | (90) | 0.0992 | 0.38 | (20) | 0.4142 | 1.59 | (19) | 0.4637 | 1.78 | (119) |
| With carryover | | | | | | | | | | | | | |
| Path-L | 1.470 | 0.0253 | 1.72 | (80) | 0.0165 | 1.12 | (20) | 0.0683 | 4.65 | (19) | 0.0747 | 5.08 | (119) |
| Well-I | 6.293 | 0.1296* | 2.06 | (80) | 0.0 | 0.00 | (20) | 0.1158 | 1.84 | (19) | 0.1712 | 2.72 | (119) |
| Geo A 632 | 26.063 | 0.1579 | 0.61 | (80) | 0.1200 | 0.46 | (20) | 0.4091 | 1.57 | (19) | 0.4547 | 1.74 | (119) |
| **Urea** | | | | | | | | | | | | | |
| Without carryover | | | | | | | | | | | | | |
| Path-L | 2.961 | 0.0378 | 1.28 | (79) | 0.0266 | 0.90 | (20) | 0.1120 | 3.78 | (19) | 0.1210 | 4.09 | (118) |
| Well-I | 9.095 | 0.1215 | 1.34 | (80) | 0.0562 | 0.62 | (20) | 0.1377 | 1.51 | (19) | 0.1920 | 2.11 | (119) |
| Geo A 632 | 31.761 | 0.4851 | 1.53 | (80) | 0.0 | 0.00 | (20) | 0.2719 | 0.86 | (19) | 0.5561 | 1.75 | (119) |
| With carryover | | | | | | | | | | | | | |
| Path-L | 2.956 | 0.0327 | 1.11 | (80) | 0.0194 | 0.66 | (20) | 0.1152 | 3.90 | (19) | 0.1214 | 4.11 | (119) |
| Well-I | 9.069 | 0.1842* | 2.03 | (80) | 0.1123 | 1.24 | (20) | 0.1201 | 1.32 | (19) | 0.2469 | 2.72 | (119) |
| Geo A 632 | 31.777 | 0.4856 | 1.53 | (80) | 0.1289 | 0.48 | (20) | 0.2085 | 0.66 | (19) | 0.5439 | 1.71 | (119) |

* *Carryover effect at α.01 level*

Fluoride oxalate plasma was used for glucose estimation. Care must be taken to ensure that plasma samples are obtained from blood samples which had the recommended amounts of anticoagulant added. High concentrations of anticoagulant resulting from inadequate filling of a specimen container could adversely affect measurement by a test or comparative method [3].

The choice and control of the comparative method represents the second major problem in the comparison of methods experiment and whereas the protocol discusses briefly the factors affecting the choice of a comparative or reference method it does not provide guidance as to the control of that method during the period of study. The data presented in Table 6 represents an attempt to provide some information about the bias of the comparative methods with reference to their overall and method means. It can be seen that the comparative method for urea shows a significant proportional error of the order of +5% when compared against overall mean values which may account in part for the significant proportional error of approximately -5% for the Kodak results against the comparative results using patient samples.

In the preparation of accuracy performance claims it is clear that calculation of bias is dependent on reliable estimates of slope and intercept and that the tolerance limits are additionally dependent on the standard error about the regression line (Syx).

Preparation of data in the manner recommended will sometimes lead to a reduction in the range of samples analysed and additionally the removal of outliers will reduce the value of Syx. For performance claims to be comparable these factors must be taken into account. These effects are discussed in detail elsewhere [8]. The protocol suggested that tolerance limits and total error be calculated only for medical decision concentrations closest to the mean of the comparative method data (x). Table 5 shows that for glucose this requirement is reasonably well fulfilled. For the medical decision concentration of 6.6 mmol/l the value of the mean of x was 7.1. However for urea the situation was less than satisfactory with a mean value of x of 7.8 and a medical decision concentration of 9.60 mmol/l. This problem has however already appeared in the literature [9] with a medical decision concentration of 1100 mg/l (6.2 mmol/l) for glucose having tolerance limits and total error quoted when the means of comparative or reference methods were 1670 mg/l (9.3 mmol/l) and 1600 mg/l (9.0 mmol/l) respectively and for a medical decision concentration of 250 mg/l (8.9 mmol/l) for urea nitrogen with a comparative method mean at 512 mg/l (18.3 mmol/l). It will be necessary to indicate how close is close if performance claims are to be of value and be comparable. The mean of the comparative method (x) should be given in an accuracy performance claim in order to avoid misunderstanding.

The proposed standard, PSEP-4, would be improved by inclusion of some basic criteria for evaluation of the comparative method against other laboratories in the form of method means. It is in this area that manufacturers are most vulnerable to claims made for or against their products by laboratories using inadequately controlled comparative or reference techniques.

**Table 5. Accuracy performance claims at selected medical decision concentrations**

| Chemistry | Medical decision level $X_C$ (mmol/l)* | Mean of X | $Y_C$** | Bias $\bar{Y}_C - \bar{X}_C$ | Tolerance limits $Y_C \pm K$ (Syx) | Est of total error KSyx ($\bar{Y}_C - \bar{X}_C$) |
|---|---|---|---|---|---|---|
| Glucose | 2.8 | | 2.6 | 0.2 | | |
| | 5.6 | | 5.4 | 0.2 | | |
| | 6.6 | 7.1 | 6.4 | 0.2 | 5.7 to 7.1 | 0.9 |
| Urea | 9.6 | 7.8 | 8.9 | 0.7 | 7.8 to 10.0 | 1.8 |

*K = Tolerance factor for Y = 0.99 p = 0.96 [10]*

** = Barnet (1968)*

*** = Based on data set $e_3$*

**Table 6. Comparison studies between Kodak and AutoAnalyzer with overall and method means for Burroughs Wellcome Quality Control sera (N = 12, mean of pairs)**

| x | y | Range (mmol/l) | Slope | SD slope | Intercept | SD intercept | Syx | Corr coeff | SDx / Syx |
|---|---|---|---|---|---|---|---|---|---|
| **Glucose** | | | | | | | | | |
| (a) mean of AA | mean of Kodak | 3.30-13.85 | 0.988 | 0.016 | -0.088 | 0.145 | 0.222 | 0.9987 | 19.2 |
| (b) overall mean | mean of Kodak | 3.40-13.77 | 0.993 | 0.019 | -0.198 | 0.179 | 0.270 | 0.9981 | 15.7 |
| (c) method mean | mean of AA | 3.37-13.72 | 1.005 | 0.014 | -0.042 | 0.129 | 0.197 | 0.9990 | 21.5 |
| (d) overall mean | mean of AA | 3.40-13.77 | 1.006 | 0.014 | -0.108 | 0.126 | 0.191 | 0.9991 | 22.1 |
| **Urea** | | | | | | | | | |
| (e) mean of AA | mean of Kodak | 5.55-24.75 | 0.953 | 0.029 | -0.889 | 0.479 | 0.663 | 0.9955 | 10.5 |
| (f) overall mean | mean of Kodak | 5.21-23.61 | 1.000 | 0.029 | -0.893 | 0.457 | 0.633 | 0.9959 | 10.5 |
| (g) method mean | mean of AA | 5.20-24.18 | 1.021 | 0.010 | 0.215 | 0.157 | 0.219 | 0.9996 | 31.2 |
| (h) overall mean | mean of AA | 5.21-23.61 | 1.049* | 0.008 | 0.006 | 0.135 | 0.186 | 0.9997 | 35.7 |

** see text for discussion of significant difference*

Because of the difficulties associated with obtaining patient samples and the labile nature of some analytes, manufacturers will always require the assistance of clinical chemistry laboratories in the establishment of performance claims, but our experience suggests that this work should not be undertaken lightly by laboratories and that manufacturers would be advised to assess the resources of any chosen site carefully before proceeding.

ACKNOWLEDGEMENTS
The authors would like to acknowledge the help of Kodak in supplying instruments and materials used in this investigation. We have valued comment from Drs R.B. Coolen and S. Reynolds and extensive secretarial help from Mrs Rosemary Jones during the preparation of this paper.

REFERENCES

[1] PSEP-2, PSEP-3, PSEP-4. Draft documents by the Instrument Evaluation Sub-committee, Evaluation Protocols Area, National Committee for Clinical Laboratory Standards, 771 E.Lancaster Avenue, Villanova, PA 19085, 1978
[2] Curme, H.G., Columbus, R.L., Dappen G.M. *et al* (1978), *Clinical Chemistry*, **24**, 1335
[3] Spayd, R.W., Bruschi, B., Burdick, B.A. *et al* (1978), *Clinical Chemistry*, **24**, 1343
[4] Kodak EKTACHEM GLU/BUN Analyser - Operators Manual (Eastman Kodak Company, Rochester NY)
[5] Wakkers, P.J.M., Hellendoorn, H.B.A., Op De Weegh, G.J. and Heerspink, W. (1975) *Clinica Chimica Acta*, **64**, 173
[6] Westgard, J.D., de Vos, D.J., Hunt, M.R., Quam, E.F., Garber, C.C. and Carey, R.N. (1978) *American Journal of Medical Technology*, **44**, 552 (1978)
[7] Davis, R.B., Thompson, J.E. and Pardue, H.L. (1978) *Clinical Chemistry*, **24**, 611 (1978)
[8] Burnett, D., Barbour, H.M. and Woods, T.F., Method Comparisons, influence of the number, distribution and range of samples on performance claims *Journal of Automatic Chemistry*, this issue, p.178.
[9] Garber, C.C., Westgard, J.O., Milz, L. *et al* (1979), *Clinical Chemistry*, **25**, 1730.
[10] Introduction to Statistical Analysis, Dixon, W.J., Massey, F.J., 3rd edition (McGraw-Hill, New York 1969), Table A-16, p.534-5

### Method comparisons, influence of the number, distribution and range of samples on performance claims

**D. Burnett, H.M. Barbour and T.F. Woods**

*Departments of Clinical Biochemistry, Queen Elizabeth II Hospital, Welwyn Garden City and St Albans City Hospital, St. Albans, Herts, UK.*

### Introduction

The previous paper [2] described two method comparison studies which followed the guidelines of the National Committee for Clinical Laboratory Standards protocol PSEP-4, comparison of methods experiment [1]. The Kodak Ektachem analytical system for urea and glucose was compared with Technicon AutoAnalyzer I methodologies. Two hundred patient samples distributed according to PSEP-4 guidelines were analysed in duplicate by the test and comparative methods. Twice the minimum recommended number of patient samples were used in order to study the effect of sample size above as well as below the recommended minimum number. The data for glucose is presented and the data modified to produce changes in the sample number, distribution and range.

The estimates of slope, intercept and standard error of the estimate of y (Syx) from linear regression analysis are used in the calculation of the tolerance limits and in estimates of total error at medical decision levels, which provide a basis for manufacturers' performance claims. This paper illustrates the way in which sample number, distribution and range could alter the manufacturers' performance claims and gives an indication of the magnitude of these effects. The methods adopted for detection of outliers in the data can also have a marked effect on the claims made.

### Materials and methods

Experimental methods and materials for glucose have been described previously [2]. The distribution of patient samples recommended for glucose analysis was Group A (<2.8 mmol/l) 10%; B (2.9-6.1 mmol/l) 40%; C (6.2-8.3 mmol/l) 30%; D (8.4-13.8 mmol/l) 10%; and Group E (>13.8 mmol/l) 10%. The information in the draft version of the PSEP-4 protocol contained a misprint and groups for glucose were given as A (10%), B (40%), C (20%, D (10%) and E (10%). In our experiment 20% of samples were collected in Group E. However, the recommended distribution and our distribution have been compared with other possible distributions for one hundred samples by data modification described below.

The equations for linear regression analysis were those given in Davies *et al* [3]. Modification of the original data base of two hundred samples analysed in duplicate by test and comparative method is described below.

### Range of samples

The results were divided into their five separate groups (A-E) and modified data sets for linear regression analysis provided by increasing range from low concentrations, A, AB, ABC, ABCD, ABCDE and from high concentrations, E, DE, CDE, BCDE, ABCDE and from mid concentrations, C, BCD, ABCDE.

